# Understanding Barriers to Guideline-Concordant Treatment in Foregut Cancer: From Data to Solutions

**DOI:** 10.1245/s10434-024-15627-9

**Published:** 2024-07-02

**Authors:** Annabelle L. Fonseca, Rida Ahmad, Krisha Amin, Manish Tripathi, Ahmed Abdalla, Larry Hearld, Smita Bhatia, Martin J. Heslin

**Affiliations:** 1 Department of Surgery, The University of Alabama at Birmingham, Birmingham, AL USA; 2 Institute for Cancer Outcomes and Survivorship, The University of Alabama at Birmingham, Birmingham, AL USA; 3 Department of Surgery, The University of South Alabama, Mobile, AL USA; 4 Kellogg School of Management, Northwestern University, Chicago, IL USA; 5 Department of Health Services Administration, The University of Alabama at Birmingham, Birmingham, AL USA

## Abstract

**Background:**

A large proportion of patients with foregut cancers do not receive guideline-concordant treatment (GCT). This study sought to understand underlying barriers to GCT through a root cause analysis approach.

**Methods:**

A single-institution retrospective review of 498 patients with foregut (gastric, pancreatic, and hepatobiliary) adenocarcinoma from 2018 to 2022 was performed. Guideline-concordant treatment was defined based on National Comprehensive Cancer Network guidelines. The Ishikawa cause and effect model was used to establish main contributing factors to non-GCT.

**Results:**

Overall, 34% did not receive GCT. Root causes of non-GCT included Patient, Physician, Institutional Environment and Broader System-related factors. In decreasing order of frequency, the following contributed to non-GCT: receipt of incomplete therapy (*N* = 28, 16.5%), deconditioning on chemotherapy (*N* = 26, 15.3%), delays in care because of patient resource constraints followed by loss to follow-up (*N* = 19, 11.2%), physician factors (*N* = 19, 11.2%), no documentation of treatment plan after referral to oncologic expertise (*N* = 19, 11.2%), loss to follow-up before oncology referral (*N* = 17, 10%), nonreferral to medical oncologic expertise (*N* = 16, 9.4%), nonreferral to surgical oncology in patients with resectable disease (*N* = 15, 8.8%), and complications preventing completion of treatment (*N* = 11, 6.5%). Non-GCT often was a function of multiple intersecting patient, physician, and institutional factors.

**Conclusions:**

A substantial percentage of patients with foregut cancer do not receive GCT. Solutions that may improve receipt of GCT include development of automated systems to improve patient follow-up; institutional prioritization of resources to enhance staffing; financial counseling and assistance programs; and development and integration of structured prehabilitation programs into cancer treatment pathways.

**Supplementary Information:**

The online version contains supplementary material available at 10.1245/s10434-024-15627-9.

Abdominal foregut cancers, i.e., cancers of the stomach, pancreas, liver, and biliary system, account for more than 20% of new cancer deaths in the United States each year.^1^ These cancers are associated with poor overall survival due to both tumor-related factors, including inherent biological aggressiveness and relative chemoresistance, as well as challenges to the delivery of care. Guideline-concordant treatment (GCT), i.e., stage-specific, standard of care treatment has been shown to improve patient outcomes in foregut cancers; however, institutional and national studies report that up to 70% of patients with foregut cancers do not receive GCT.^2–8^

A large percentage of patients with foregut cancer present with metastatic disease, where systemic therapy is the mainstay of treatment. In patients with anatomically resectable, nonmetastatic cancer, multimodal therapy with curative-intent surgical resection and chemotherapy is generally recommended. Optimal treatment requires coordinated, highly specialized multidisciplinary care, often at tertiary care centers. In addition to cancer-related deconditioning, technically complex operations associated with morbidity and deconditioning, and challenging chemotherapy regimens pose challenges to patients.

While numerous studies have demonstrated that sociodemographic variables, such as race, socioeconomic status, and rurality impact receipt of GCT,^5,6,9–11^ these studies do not provide information about individual barriers to accessing GCT. Disparities in the receipt of GCT in foregut cancer are likely caused by multiple unmeasured barriers along the cancer care continuum, several of which are potentially modifiable. The objective of this study was to identify the underlying barriers to receipt of GCT among patients with foregut cancers treated at an academic center in the Deep South through a root cause analysis approach.

## Methods

### Study Population

A retrospective review was performed of electronic health records of patients with pancreatic, gastric, and hepatobiliary adenocarcinoma treated at the University of South Alabama Health System between January 2018 and December 2022. Patients were identified based on the International Classification of Disease (ICD) codes associated with the specific cancers: C25.0-C25.3, C25.7–C25.9 (pancreatic), C16.0–C16.9 (gastric) and C22.1, C22.7–C22.9, C23, C24.0, C24.1, C24.8, C24.9 (hepatobiliary). This study was approved by the institutional review board at the University of South Alabama.

The University of South Alabama serves as the safety-net hospital and main tertiary-referral center for southern and central Alabama and the adjoining areas of the Gulf coast, an area that is home to more than 5 million people, with a large rural and racial minority population.^12^

### Data Collection

Manual chart abstraction from the electronic health record (EHR) included sociodemographic factors, cancer-specific variables, and access-related variables. Sociodemographic data collected included age, race, relationship status, and residence in a rural location. Cancer-specific data collected included type of cancer, clinical stage, pathologic stage, cancer-specific staging imaging, curative-intent surgical treatment, systemic therapy, and radiation therapy. Access-related data collected included insurance status, comorbidity status assessed by using the Charlson Comorbidity Index, presence of a primary care physician, prior screening behavior (age- and guideline-appropriate colonoscopy and mammogram screening), where the cancer was diagnosed (in the outpatient setting or in the emergency department (ED) without preexisting workup initiated by patient’s physician in the outpatient setting), and ED utilization after establishment of cancer diagnosis. Additionally, data regarding time to access oncologic care were collected for significant timepoints, i.e., time from symptoms to start of workup, time from diagnosis to being seen by relevant oncologic expert, and time from oncology appointment to start of treatment. Documented reasons for non-receipt of therapy were collected. Area Deprivation Index (ADI) score, a validated neighborhood-level composite index that reflects the neighborhood deprivation relative to the remainder of the state, was calculated for each patient by using the ADI mapping atlas and patient addresses and grouped into tertiles: low deprivation (low ADI; least disadvantaged), and intermediate deprivation and high deprivation (high ADI; most disadvantaged) as described in a prior publication.^8^

### Definition of Guideline-Concordant Treatment

Guideline-concordant therapy (GCT) was defined based on National Comprehensive Cancer Network guidelines and varied based on type and stage of cancer. In the case of metastatic disease, GCT was defined as receipt of systemic therapy, unless there was documentation of clinical status that precluded receipt, or a palliative care discussion and patient desire not to receive therapy. In the case of nonmetastatic disease, GCT varied based on assessment of anatomic resectability. In patients with nonmetastatic stage I and II pancreatic adenocarcinoma, GCT was defined as receipt of chemotherapy and curative-intent surgical resection in any sequence, unless there was documentation of comorbidities that precluded resection, or progression of disease on chemotherapy. For stage III pancreatic adenocarcinoma, GCT was defined as receipt of chemotherapy, with or without curative-intent surgical resection or radiation therapy. In patients with nonmetastatic gastric adenocarcinoma, GCT was defined as receipt of endoscopic or surgical resection (for T1a and T1b tumors without nodal or other involvement), or chemotherapy and curative-intent surgical resection, unless there was documentation of comorbidities that precluded resection, or progression of disease on chemotherapy. In patients with nonmetastatic cholangiocarcinoma, GCT varied based on location and surgeon assessment of resectability: for intrahepatic and extrahepatic cholangiocarcinoma determined to be resectable, GCT was defined as curative-intent surgical resection (if R0 resection were achieved before 2020) or curative-intent surgical resection and systemic therapy (2020 onwards, and in all patients without an R0 resection); for perihilar cholangiocarcinoma, GCT was defined as curative-intent surgical resection with or without radiation, with or without systemic therapy, or systemic therapy with or without radiation. For patients with resectable gallbladder adenocarcinoma, GCT was defined as curative-intent surgical resection (if R0 resection were achieved before 2020) or curative-intent surgical resection and systemic therapy (2020 onwards, and in all patients without an R0 resection). For unresectable gallbladder adenocarcinoma, GCT was defined as systemic therapy with or without radiation. The type- and stage-based definition of GCT has been summarized in Supplementary Table 1. In all cases GCT was determined by review of medical records to account for adherence to NCCN type- and stage-appropriate guidelines, with consensus regarding definition of GCT achieved by two surgical oncologists.

### Root Cause Analysis Methodology

Root Cause Analysis (RCA) is a systematic process to establish the underlying causes of a problem or event to identify appropriate solutions and typically includes the following steps: (1) Define the problem; (2) Gather information; (3) Identify causal factors; (4) Identify root causes; and (5) Propose and prioritize solutions or corrective actions.^13,14^ Several RCA techniques exist, including Pareto charts, the five whys method, fault tree analysis, and the Ishikawa cause and effect diagram (fishbone model), all of which follow a structured approach to determine the reasons that a particular problem has occurred.

The problem was defined as non-receipt of GCT among patients with foregut cancer. Medical records of all patients who did not receive GCT were reviewed in their entirety. Data regarding onset of symptoms, workup, diagnosis, access to oncologic expertise, treatment plans, treatment initiation, and adherence were extensively reviewed. The fishbone model was used to establish the main contributing factors. Major categories representing potential causes for nonreceipt of GCT were identified and selected. Under each category, specific causes or subcauses pertinent to the problem were detailed based on analysis of the collected data. Consensus regarding the underlying causes was achieved by three coauthors. The information from the 170 cases with nonreceipt of GCT was synthesized and used to populate the Fishbone model with common themes, recurring issues, and shared factors contributing to nonreceipt of GCT.

## Results

A total of 498 patients with foregut cancer were seen at the institution during the study period, of whom 170 patients (34.1%) did not receive GCT. Receipt of GCT varied based on underlying cancer: 40.6% in hepatobiliary, 33.9% in pancreatic, and 28.3% in gastric adenocarcinoma. Of the 170 patients who did not receive GCT, 50% were male, 53% were White, 43% were married, and the majority (58%) were between the ages of 55 and 74 years. Most patients (65%) were insured through Medicare, followed by private insurance (21%). Seventeen percent of patients lived in rural locations. The majority of patients (55%) had their workup initiated in the ED, and more than 70% of patients did not have prior cancer screening in the form of guideline-appropriate colonoscopy or mammography screening. Median (interquartile range [IQR] time from symptoms to start of workup was 8.8 (range 4.3–16.4) weeks, time from diagnosis to first oncology appointment was 4.6 (range 1.6–6.6) weeks, and time from oncology appointment to start of cancer treatment was 11 (range 3.7–18.2) weeks.

Compared with patients who received GCT, patients who did not receive GCT were more likely to be older (29% vs. 16% of patients ≥ 75 years, *p* = 0.003), Black (42% vs. 23%, *p* < 0.0001), unmarried (57% vs. 40%, *p* < 0.0001), and living in an area of high deprivation (47% vs. 20%, *p* < 0.0001). Patients who did not receive GCT also were more likely to have their workup initiated in the ED (55% vs. 41%, *p* = 0.003), not to have prior age-appropriate cancer screening (27% vs. 39%, *p* = 0.004), and to have higher ED utilization after their cancer diagnosis (20% vs. 13%, *p* = 0.03), indicating continued issues with access to care. Baseline sociodemographic and other relevant variables are summarized in Table 1. Time to care was significantly longer for patients who did not receive GCT (Table 2).

**Table 1:** Baseline demographic variables

Variable	Overall cohort	Patients receiving non-GCT; *N* (%)	Patients receiving GCT; *N* (%)	*p*
*Age (years)*				
<55	79 (15.9%)	22 (12.9%)	57 (17.4%)	0.003
55–74	315 (63.2%)	98 (57.7%)	217 (66.2%)	
75+	104 (20.9%)	50 (29.4%)	54 (16.5%)	
*Sex*				
Male	274 (55%)	85 (50%)	189 (57.6%)	0.1
Female	224 (45%)	85 (50%)	139 (42.4%)	
*Race*				
White	327 (65.7%)	90 (52.9%)	237 (72.3%)	<0.0001
Black	145 (29.1%)	71 (41.8%)	74 (22.6%)	
Other	26 (5.2%)	9 (5.3%)	17 (5.2%)	
*Presence of primary care physician*				
No	185 (37.2%)	52 (30.6%)	58 (17.7%)	0.002
Yes	313 (62.8%)	118 (69.4%)	270 (82.3%)	
*Workup initiated by*				
Emergency department	227 (45.6%)	93 (54.7%)	134 (40.9%)	0.003
Primary care physician	271 (54.4%)	77 (45.3%)	194 (59.2%)	
*Insurance status*				
Medicare	284 (57%)	111 (65.3%)	173 (52.7%)	0.06
Private	128 (25.7%)	36 (21.2%)	92 (28%)	
Medicaid	33 (6.6%)	11 (6.5%)	22 (6.7%)	
Uninsured	30 (6%)	8 (4.7%)	16 (4.9%)	
Veterans Affairs	23 (4.6%)	4 (2.4%)	25 (7.6%)	
*Prior cancer screening*				
No	324 (65.1%)	125 (73.5%)	199 (60.7%)	0.004
Yes	174 (34.9%)	45 (26.5%)	129 (39.3%)	
*Emergency department (ED) visits after diagnosis*				
No ED visits	269 (54%)	82 (48.2%)	187 (57.0%)	0.03
1–2 ED visits	153 (30.7%)	54 (31.8%)	99 (30.2%)	
3+ ED visits	76 (15.3%)	34 (20%)	42 (12.8%)	
*Residence in a rural county*				
No	426 (85.5%)	142 (83.5%)	284 (86.6%)	0.3
Yes	72 (14.5%)	28 (16.5%)	44 (12.4%)	
*Marital status*				
Married	270 (54.2%)	73 (43%)	197 (60.1%)	<0.0001
Unmarried	228 (45.8%)	97 (57%)	131 (39.9%)	
*Area Deprivation Index*				
Low deprivation	207 (41.6%)	48 (28.2%)	159 (48.5%)	<0.0001
Intermediate deprivation	147 (29.5%)	43 (25.3%)	104 (31.7%)	
High deprivation	144 (28.9%)	79 (46.5%)	65 (19.8%)	

*GCT* guideline-concordant treatment

**Table 2:** Timeliness of care based on guideline concordant treatment

	Patients receiving non-GCT; *N* (%)	Patients receiving GCT; *N* (%)	*p*
*Time from symptoms to start of work-up in weeks*			
Median (IQR)	8.8 (4.3–16.4)	4.6 (2.5–8.7)	<0.0001
*Time from diagnosis to oncology appointment in weeks*			
Median (IQR)	4.6 (1.6–6.6)	2.6 (1.1–4.8)	<0.0001
*Time from oncology appointment to complete staging in weeks*			
Median (IQR)	0.5 (0.1–5.4)	0.1 (0.1–1.5)	0.21
*Time from oncology appointment to start of treatment in weeks*			
Median (IQR)	11 (3.7–18.2)	2.9 (1.5–5)	<0.0001

*IQR* interquartile range; *GCT* guideline-concordant treatment

**Fig. 1:**
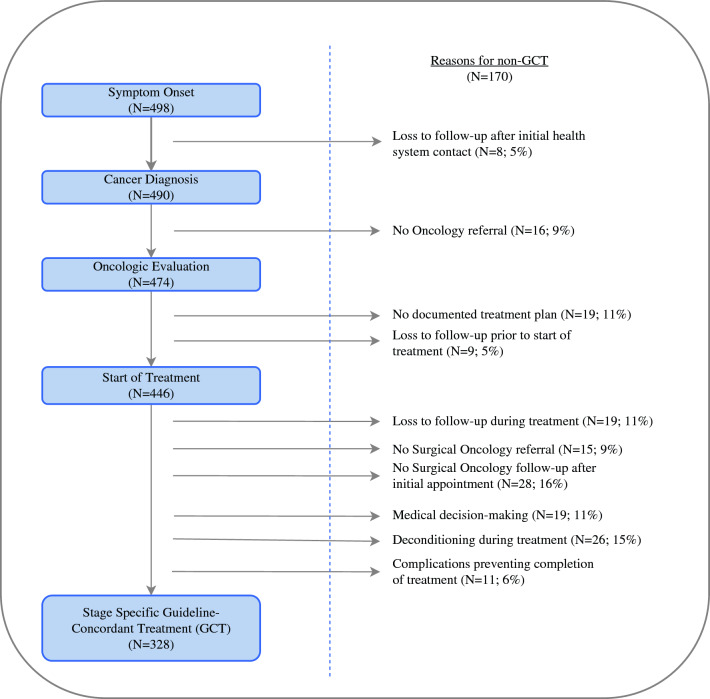
Barriers to receipt of guideline-concordant treatment along the cancer care continuum

Multiple barrier points to receipt of GCT were noted, resulting in drop-out of patients along the cancer care continuum (Fig. 1). In decreasing order of frequency, the following reasons contributed to non-GCT: receipt of incomplete therapy because of lack of follow-up with surgical oncologist after initial appointment (*N* = 28, 16.5%), deconditioning on chemotherapy resulting in nonreceipt of surgical therapy (*N* = 26, 15.3%), delays in care because of patient resource constraints followed by loss to follow-up after receipt of some therapy (*N* = 19, 11.2%), physician factors, including nondiagnosis because of provider knowledge, or specialist knowledge of or adherence to guidelines (*N* = 19, 11.2%), no documentation of treatment plan after referral to oncologic expertise (*N* = 19, 11.2%), loss to follow-up before oncology referral without any record of health system attempt to reestablish care (*N* = 17, 10%), nonreferral to any oncologic expertise (*N* = 16, 9.4%), nonreferral to surgical oncologic expertise in patients with resectable disease (*N* = 15, 8.8%), and complications during treatment preventing completion of treatment (*N* = 11, 6.5%).

**Fig. 2:**
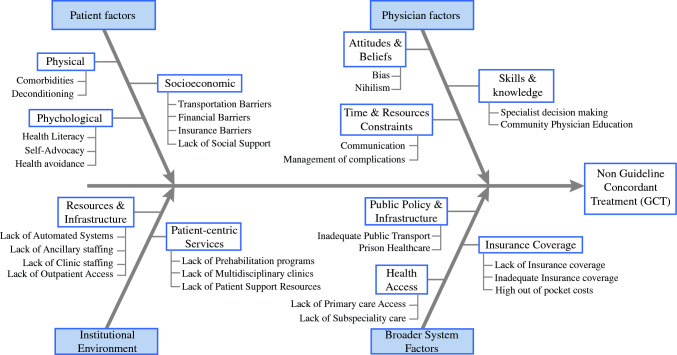
Fishbone model with root causes of nonguideline concordant treatment among patients with foregut cancer

The Fishbone model demonstrating root causes of non-GCT is demonstrated in Fig. 2. Factors related to non-GCT include patient, physician, institutional environment, and broader system factors. Patient factors included transportation and financial barriers, lack of social support, health literacy, preexisting comorbidities, deconditioning, and self-advocacy. Physician factors included medical education or decision-making, time-related barriers, including lack of appropriate referrals, follow-up and communication, as well as bias and nihilism. Examples of physician factors included inadequate or incorrect operations (e.g., liver resection without portal lymphadenectomy in the case of known cholangiocarcinoma, cholecystectomy alone for T3 gallbladder adenocarcinoma without additional curative-intent resection in the absence of contraindications), physician medical knowledge (e.g., new onset diabetes, weight loss, and jaundice not recognized as being suspicious for pancreatic cancer, biliary strictures thought to be benign without adequate workup), and physician adherence to guidelines (e.g., surgical resection alone for T2+ gastric cancer without referral to medical oncologist). Institutional environment factors were important in several instances of non-GCT, in particular, lack of automated systems for scheduling and patient follow-up (e.g., follow-up appointments not scheduled, patient appointments not being rescheduled in case of missed appointments), institutional resources for staffing (e.g., physical therapy, nutrition, navigation, social services), and patient support (e.g., financial support for co-pays and transportation). Broader system factors included insurance related factors (lack of insurance, under-insurance, high out-of-pocket costs), as well as accessibility-related factors (lack of primary care access, lack of subspecialty care in rural areas resulting in long distances to access care, and lack of public transportation). Overall, patient factors, physician factors, institutional environment factors, and broader system factors contributed to 34%, 73%, 78%, and 31% of non-GCT.

Non-GCT often was a function of multiple intersecting patient, physician, and health system factors: e.g., hospital admission for a post-procedural complication resulting in a missed appointment and lack of rescheduling resulting in loss to follow-up; deconditioning during chemotherapy as a function of preexisting comorbidities, lack of physical therapists, and lack of a structured prehabilitation program to prevent deconditioning during neoadjuvant chemotherapy.

## Discussion

**Fig. 3:**
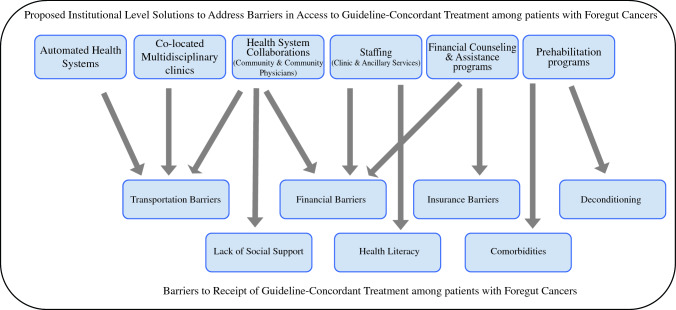
Proposed institutional-level solutions to improve receipt of guideline-concordant treatment among patients with foregut cancer

This study examined the underlying causes of non-GCT in patients with foregut cancer treated at a safety-net hospital and tertiary care academic center in Southern Alabama. A detailed analysis of individual patient medical records and a structured approach demonstrated multiple contributory and often intersecting patient, physician, institutional environment, and broader system factors. Determining the underlying root causes allows us to propose and develop targeted solutions that could prevent future instances of non-GCT. While several of these factors may require policy level solutions to address them most comprehensively, several institutional level solutions may address some of these factors (Fig. 3). Table 3 summarizes proposed solutions to address these barriers. A more detailed table summarizing underlying factors, patient-specific examples of each of these factors and proposed solutions is available in Supplementary Table 2.

**Table 3:** Proposed solutions to address physician, institutional and systems-level factors

Proposed solution	Barrier addressed
*Patient-level*	
Institutional prioritization of primary care access in the community	Outpatient healthcare access
Collaboration with existing primary care resources to increase capacity, establish, and support referral networks	Outpatient healthcare access
Improved access to outpatient care for patients in active treatment, e.g., infusion clinics, symptom management clinics	Outpatient healthcare access
Tailored prehabilitation programs with automatic enrollment for all patients: nutrition advice, graded exercise regimens	Comorbidities Deconditioning during treatment
Institutional collaboration with community health workers, foundations, and community-based organizations to create support networks that may provide patients with transportation and other support services.	Transportation barriers Social support barriers Financial barriers
Financial counselling and assistance programs: Financial assistance prior to cancer diagnosis Transportation Insurance	Financial barriers Transportation barriers Insurance barriers
Prioritization of staffing of ancillary services: Navigation Social services	Financial barriers Transportation barriers Insurance barriers Health literacy
Prioritization of clinic staffing Front office Scheduling	Communication barriers Health literacy
Automated systems Increase follow-up Patient reminders for appointments, tests Flag patients for prehabilitation	Health literacy Communication barriers Comorbidities Deconditioning
Co-located multidisciplinary clinics to streamline cancer care	Transport barriers
Community engagement	Health literacy Self-advocacy
Development of education programs and distribution of education materials at fifth grade level to improve health literacy and empower patients to advocate for their cancer care.	
*Physician-level*	
Development of electronic health record (EHR) prompts to improve compliance with medical guidelines	Physician knowledge/education
Multidisciplinary discussion for all new cancer patients	
Reflexive surgical referral for all patients with nonmetastatic disease	
Development of protocols to manage procedure-related complications promptly to minimize impact on cancer treatment and outcomes.	
Community physician education	Weaker ties with community and community physicians
Collaboration with local healthcare providers to establish and support referral networks and provide education	
Documentation of care plans, referrals	Physician communication
*Broader system-level*	
Collaborate with policymakers and healthcare stakeholders to address systemic challenges	Insurance access barriers, including inadequate coverage Inadequate public transport
Advocate for policy changes to expand insurance coverage	Insurance access barriers
Institutional collaboration to improve healthcare for vulnerable populations including those in prison settings	Prison healthcare

We focus a priori on health system level solutions that may be the most impactful in addressing the underlying root causes of non-GCT. The development of automated systems at the institutional electronic health record (EHR) level could greatly increase receipt of GCT through multiple mechanisms. Automated scheduling of follow-up appointments may have prevented dropout in the case of 47% of the patients who did not receive GCT. Automated systems may also improve compliance with GCT at the physician level with prompts for appropriate staging, and referral to medical or surgical oncologic expertise that may have impacted receipt of GCT in 18% of the patients. Automated EHR prompts that flag patients with new cancer diagnoses for automatic enrollment in prehabilitation programs could prevent deconditioning during receipt of neoadjuvant systemic therapy, thereby allowing more patients to be considered for curative-intent surgical resection if otherwise appropriate based on stage and underlying tumor biology. Structured prehabilitation programs have been shown to be beneficial in patients with hepatobiliary, pancreatic, and other abdominal malignancies and have shown benefits, including improved physiology, improved quality of life, and decreased morbidity.^15–17^ Providing this program to all patients with automatic enrolment will likely increase the number of patients that become candidates for surgical resection. Programs such as this may have improved receipt of GCT in our patient cohort by 15%.

Institutional prioritization of staffing at patient-facing levels including scheduling and clinic staffing, social work, care coordination, and navigation would not only allow for improved patient experience, but also greatly enhance cancer care equity. Navigation, a patient-centered intervention that serves to improve the delivery of cancer care has been shown to improve health equity across the cancer care continuum.^18–21^ Locally informed patient-centered navigation programs may address financial and insurance barriers by identifying resources for insurance coverage and facilitating access to community-based food and housing services, as well as self-advocacy and health literacy barriers through bidirectional communication and serving as an intermediary between physician teams and the patient. Navigation also may address transportation and other access barriers through the coordination of care and care teams by facilitating referrals to primary care and ensuring subspecialty appointments are scheduled to be respectful of the time and resources of patients and caregivers.

While a retrospective analysis of our data does not allow us to establish the percentage of patients who would benefit from institutional prioritization of financial counseling and assistance programs, 21% of the patients who did not receive GCT were lost to follow-up before (10%) or after (11%) the start of treatment. It is likely that financial assistance could have improved GCT in this patient population. Once a diagnosis of cancer is made, patients may become eligible for financial aid through the cancer center or other resources; however, there is a dearth of funds to assist patients before a cancer diagnosis is established. Establishment of a cancer diagnosis may require an extensive and financially challenging workup, including imaging, repeat biopsies, and multiple appointments with gastrointestinal and oncologic expertise, all of which pose substantial challenges to patients with financial barriers, lack of social support, or who have to travel long distances to access care. Maintenance of institutional resources for workup of patients may have benefited the 5% of patients who were lost to follow-up before establishment of a cancer diagnosis.

Several other institutional endeavors may improve access and adherence to GCT through multiple mechanisms. Co-located multidisciplinary cancer care clinics have been shown to provide improvements in quality of care across multiple levels, including shorter time to treatment, increased likelihood of appropriate staging, and receiving stage-appropriate GCT across several cancer types.^22–24^ In addition to improving medical decision-making, this approach would address patient transport barriers and may address other physician barriers, such as bias and nihilism. In locations where physical co-location of clinics is not feasible, telehealth may provide options that allow for multidisciplinary medical decision-making. Telehealth may allow for development of collaborations with physicians in the community through virtual tumor board discussions and support existing collaboration with community primary care providers as well as other supportive care and prehabilitation clinics.

Medicaid expansion, if adopted in Alabama, could provide an avenue for institutions to fund interventions designed to improve access to GCT. By reducing the number of uninsured patients requiring care, Medicaid expansion could reduce the burden of uncompensated and undercompensated care faced by institutions. The Affordable Care Act provides for expanded Medicaid eligibility to adults with incomes at or below 138% of the federal poverty limit in participating states and has been shown to be associated with improved receipt of cancer care in pancreatic and other foregut cancers.^25–30^ Medicaid expansion in Alabama is projected to allow coverage of more than 200,000 uninsured nonelderly adults, i.e., 49% of the state’s uninsured nonelderly adult population, including 79% of childless adults, a group that has been historically excluded from Medicaid eligibility.^31,32^ Medicaid expansion has been shown to have the highest impact on safety-net hospitals and could provide critical financial support to expand patient-centered services designed to improve receipt of GCT in patients with foregut cancers at these institutions.^33^

Central to reducing cancer health disparities is the recognition that solutions must extend beyond the clinic and hospital walls. Collaborations with communities are of paramount importance as they offer unique insights into the social determinants of health that often underlie health disparities. By engaging with all the relevant stakeholders: patients, caregivers, and other community stakeholders, health systems may be able to develop targeted interventions that address the specific needs of the community they serve.

This study has several limitations that must be considered. This is a single-institution study in a southern state, and its results may not be generalizable to other health systems or other areas of the country. Nonetheless, the underlying root causes of non-GCT likely exist in other safety-net hospitals and tertiary care centers, and as such the proposed solutions may have applicability beyond our institution. Conversely, the key strength of this study is depth and granularity of data obtained through detailed review of EHR, which may not be available in multi-institutional studies or larger databases. GCT is nuanced and can be challenging to identify in a retrospective fashion, however, every effort was made to define GCT as comprehensively as possible, based on detailed review of the EHR. Medical records were completely reviewed in each case of non-GCT in order to identify the underlying root causes and propose solutions. However, it is not certain that adoption of these solutions would have resulted in GCT. Given the complexity of factors contributing to non-GCT, improvement in receipt of GCT will likely require a combination of solutions and interventions targeting multiple barriers at various levels.

## Conclusions

This study provides valuable insight into the multiple intersecting barriers that contribute to non-GCT in patients with foregut cancer. The fundamental purpose of our healthcare systems must be to provide high-quality, equitable care to all patients. By identifying and understanding the root causes of non-GCT, we can develop targeted solutions to improve the receipt of GCT. The complexity of factors contributing to non-GCT mandates that health systems adopt a multi-pronged approach targeting multiple barriers at various levels, including the development of automated systems, patient-centered interventions, and collaborations with the community. By implementing these strategies, we believe that we can improve the delivery of high quality, guideline-concordant cancer care on a broader scale. This requires a commitment to self-evaluation and adaptation at the health system level, along with ongoing collaborations with patients, communities, and healthcare professionals.

### Supplementary Information

Below is the link to the electronic supplementary material.

Supplementary file1 (DOCX 21 KB)
