# Supplementary material for: Understanding Barriers to Guideline-Concordant Treatment in Foregut Cancer: From Data to Solutions
Source: Ann Surg Oncol. 2024 Jul 2;31(9):6007–16. doi: 10.1245/s10434-024-15627-9 (PMC11300473; doi:10.1245/s10434-024-15627-9)
Supplement: Supplementary file 1 — Supplementary file1 (DOCX 21 KB) [file 10434_2024_15627_MOESM1_ESM.docx]

**Table 1: Definition of Guideline-Concordant Treatment**

| Type of Cancer | Stage | Guideline-Concordant Treatment |
| --- | --- | --- |
| Pancreatic Adenocarcinoma | 1, 2 | Receipt of chemotherapy and curative-intent surgical therapy in any sequence (Exceptions- documentation of comorbidities that precluded resection, or progression of disease on chemotherapy) |
|  | 3 | Receipt of systemic chemotherapy +/- curative-intent surgical resection +/- radiation (Exception- documentation of comorbidities that precluded receipt) |
|  | 4 | Receipt of systemic chemotherapy (Exceptions- documentation of clinical status that precluded receipt, or a palliative care discussion and patient desire to not receive therapy) |
| Gastric adenocarcinoma | 1 | Endoscopic resection (T1a); curative-intent surgical resection (T1b) +/- systemic chemotherapy (T2) |
|  | 2, 3 | Receipt of chemotherapy and surgical therapy in any sequence (Exceptions- documentation of comorbidities that precluded resection, or progression of disease on chemotherapy) |
|  | 4 | Receipt of systemic chemotherapy (Exceptions- documentation of clinical status that precluded receipt, or a palliative care discussion and patient desire to not receive therapy) |
| Cholangiocarcinoma | 1 | Curative-intent surgical resection (if R0 resection were achieved prior to 2020) or curative-intent surgical resection and systemic therapy (2020 onwards, and in all patients without an R0 resection); Exception- documentation of unresectability or comorbidities that precluded resection |
|  | 2, 3 | If resectable: Curative-intent surgical resection (if R0 resection were achieved prior to 2020) or curative-intent surgical resection and systemic therapy (2020 onwards, and in all patients without an R0 resection)  If unresectable: Systemic therapy +/- radiation |
|  | 4 | Receipt of systemic chemotherapy (Exceptions- documentation of clinical status that precluded receipt, or a palliative care discussion and patient desire to not receive therapy) |
| Gallbladder adenocarcinoma | 1 | T1a- gallbladder resection; T1b- gallbladder resection with partial hepatectomy and portal lymphadenectomy |
|  | 2, 3 | Curative-intent surgical resection i.e., gallbladder resection with partial hepatectomy and portal lymphadenectomy (if R0 resection were achieved prior to 2020) or curative-intent surgical resection and systemic therapy (2020 onwards, and in all patients without an R0 resection), OR systemic chemotherapy and curative-intent surgical resection (Exception- documentation of comorbidities that precluded resection, or progression of disease on chemotherapy) |
|  | 4 | Receipt of systemic chemotherapy (Exceptions- documentation of clinical status that precluded receipt, or a palliative care discussion and patient desire to not receive therapy) |

**Table 2: Summary of Factors underlying Non-receipt of Guideline concordant treatment and Proposed Solutions**

| **Factor** | **Example** | **Proposed Solution** |
| --- | --- | --- |
| **Patient** |  |  |
| Pre-existing comorbidities | Procedural complication in patient with multiple pre-existing comorbidities resulted in her being unable to receive any cancer therapy | Improved access to outpatient care, tailored prehabilitation programs with nutritional support and graded exercise regimens offered to all patients. |
| Deconditioning | Patient unable have surgical resection due to deconditioning during chemotherapy |  |
| Inadequate financial resources | Patient missed staging imaging and appointments due to inability to cover out of pocket costs | Institutional financial counselling and assistance programs.  Offering information about programs to all patients. |
| Inadequate social support | Patient missed chemotherapy appointments because of no one to drive them to and from their appointments | Institutional collaboration with community health workers, foundations, and community-based organizations to create support networks that may provide patients with transportation and other support services. |
| Transportation barriers | Patient did not attend surgical appointment due to transport distance and logistics |  |
| Health Literacy | Patient was told by primary care physician there was no treatment for pancreatic cancer and did not seek other opinion | Community engagement to provide patient education.  [Development of education programs and distribution of education materials at 5^th^ grade level to improve health literacy and empower patients to advocate for their cancer care] |
| Self-advocacy | Patient with resectable pancreatic cancer referred to hospice wanted to explore treatment options but was not able to |  |
| Health Avoidance | Patient with increasing symptoms and a family history of cancer did not seek medical attention due to anxiety |  |
| **Physician** |  |  |
| Bias | Patient with pancreatic cancer not referred to oncologic expertise due to concern for patient resources | Physician education |
| Nihilism | Patient with resectable pancreatic cancer referred to hospice without appointment with medical or surgical oncology | Physician education |
| Community Physician Education | New onset diabetes, weight loss and jaundice not recognized as pancreatic cancer | Physician education  Collaboration with local healthcare providers |
| Specialist medical decision making | Total gastrectomy performed for gastric cancer without endoscopic or PET staging and without referral to medical oncology either before or after surgical resection | Multidisciplinary discussion for all new cancer patients |
| Management of complications | Cholangitis following stent occlusion not promptly managed resulting in sepsis | Establish protocols to manage procedure related complications promptly to minimize impact on cancer treatment and outcomes. |
| Communication | Patient unaware of scheduled procedure and physician medical plan | Increase staffing.  Decrease demands on physician time.  Physician education |
| **Institutional Environment** |  |  |
| Lack of automated systems   1. Staging 2. Clinic appointments | 1. Incomplete Staging  2. Patient missed scheduled clinic appointment due to hospital admission and was not rescheduled | Hospital investment in automated systems for patient follow-up.  Using electronic health record prompts to improve compliance with medical guidelines |
| Lack of patient support resources | Patient missed several chemotherapy appointments due to cost and logistics associated with transport | Institutional financial counselling and assistance programs.  Offering information about programs to all patients. |
| Lack of outpatient access | Patient with chemotherapy related dehydration being unable to communicate with care team resulting in ED admission and hospitalization for prolonged period | Institutional prioritization of resources to enhance clinic staffing. |
| Lack of staffing   1. Clinic support staff 2. Social workers 3. Navigators 4. Physical therapists 5. Dieticians | Patient who was told their insurance would not cover treatment missed appointment and treatment, and was not informed of other resources that may have covered treatment | Institutional allocation of resources to enhance staffing levels of clinic support and ancillary support services. |
| Lack of structured prehabilitation programs | Patient became increasingly deconditioned during chemotherapy | Development of structured prehabilitation programs with automatic enrollment of all appropriate cancer patients. |
| Lack of multidisciplinary clinics | Patient missed surgical appointment because they were unable to travel to facility on different days | Development of co-located multidisciplinary clinics to streamline cancer care and improve patient experience. |
| **Broader System** |  |  |
| Inadequate public transport | Patient did not receive chemotherapy because of inability to arrange for transport. | Prioritize development of institutional resources for transportation.  Institutional collaboration with foundations and community-based organizations to create transport support networks. |
| High out of pocket costs | Patient with resectable pancreatic cancer referred to hospice without appointment with medical or surgical oncology | Institutional financial resources (and provide information to all patients).  Collaborate with policymakers and healthcare stakeholders to address systemic challenges such as inadequate public transport and high out-of-pocket costs |
| Lack of primary care access | Patient with gastric cancer was seen at multiple emergency rooms over 6 months for blood transfusion, however was unable to obtain oncology appointment | Institutional prioritization of primary care access in the community.  Collaboration with existing primary care resources to increase capacity. |
| Lack of subspecialty care in remote areas | Patient was told by primary care physician that there was no treatment for pancreatic cancer and did not seek other opinion; no oncology expertise locally | Institutional prioritization of primary care access in the community.  Collaboration with existing primary care resources to increase capacity. |
| No insurance coverage | Patient did not have work-up for gastric cancer due to lack of insurance | Institutional resources for work up of patients.  [existing resources usually cover patients after a cancer diagnosis is made] |
| Inadequate insurance coverage | Patient did not complete course of therapy because insurance stopped covering treatment | Advocate for policy changes to expand insurance coverage |
| Inadequate prison healthcare | Patient with pancreatic cancer did not receive chemotherapy because of lack of transportation to appointments while in prison | Institutional collaboration to improve healthcare for vulnerable populations including those in prison settings. |
